# Puerperal Infection—Etiology and Results

**Published:** 1892-01

**Authors:** R. R. Kime

**Affiliations:** Lecturer on Diseases of Women, Southern Medical College, Atlanta, Ga.


VOL. VIII. JANUARY, 1892. No. 11.

TO CONTRIBUTORS.

Articles for publication must reach this office not later than 15th inst.; and are expected to be published exclusively in this JOURNAL. Extra copies of JOURNAL furnished contributors when requested. Reprints by special arrangement. Publication of an article does not necessarily imply endorsement of the views therein expressed.

ATLANTA MEDICAL AND SURGICAL JOURNAL, Post-office box 431.

### Original Communications.

### PUERPERAL INFECTION.—ETIOLOGY AND RESULTS.*

BY R. R. KIME, M. D,
Lecturer on Diseases of Women, Southern Medical College, Atlanta, Ga.

I have selected the term "puerperal infection," as used by Garrigues[1], of New York, as more appropriate, definite and comprehensive than "puerperal fever," "septicæmia," "septic infection" and other terms that have been used to designate abnormal conditions occurring during the puerperium as a result of infection. We find the terms used interchangeably by different authors, as if they designated one and the same condition as to origin. In this article we shall consider all infections occurring

*Written for Tri-State Medical Association.

[1] Cyclopædia of Obstetrics.—Mann.

during the puerperal state under the general term of "puerperal infection," which may be divided into,

Puerperal infection:
- Putrid infection—mild cases.
- Putrid intoxication—severe cases.
- Septic infection—mild cases.
- Septic intoxication—severe cases.

As a basis for the above division of puerperal infection, we quote, "None of the pus-generating cocci cause what is commonly called putrescence."[2]

"Decomposition of tissues accompanied by the production of foul odors is always due to the fermentative action of divers forms of elongated bodies, called bacilli or bacteria."[3]

"The chief significance of the fungi *staphylococcus pyogenes*, *aurens* and *albus*, and *streptococcus pyogenes*, is that they give to pus a septic power, which pus free from these elements does not possess."[4]

"Koch inoculated mice with putrid blood and meat infusions, five drops usually killing the mice in four to eight hours. From examination he concluded the poisoning and death was due to a chemical substance called sepsin."

"Klebs differentiates between septicæmia and pyæmia, although he claimed that putrid intoxication and septic infection were the same. Koch injected mice with blood that had putrefied for a few days only; five drops killed in a short time. One minim often produced no morbid symptoms. One-third of the animals injected with one to two drops in twenty-four hours began to manifest morbid symptoms and died in forty to sixty hours; no inflammation at seat of infection; no bacteria in the blood or internal organs, and could not be transmitted to other mice by inoculation. While one-tenth drop of œdema fluid and blood from mice dying of sepsis produced same symptoms as in the putrid inoculations, and death. The virus could be transmitted and propagated indefinitely. Small bacilli alone appeared in the blood constantly and in large numbers."[5]

---

[2] Cyclopædia of Surg., Vol. 1, p. 92.
[3] Gerster.—Rules of Aseptic and Antiseptic Surgery.
[4] Wyeth.—Text-book on Surgery, p. 62.
[5] Senn's Principles of Surgery, p. 304.

"The general symptoms which accompany all suppurative affections represent, etiologically and clinically, a form of sepsis, which differs in its intensity according to quantity of pus-microbes, or their ptomaïnes, which reach the general circulation. Besser is of the opinion that the streptococcus of suppuration is the most frequent cause of sepsis."[5]

"From these considerations it becomes evident that the essential bacterial cause of septicæmia is variable, and that the disease represents a general febrile condition, which is brought about by the absorption from a local focus of different toxines from as many microbes. As the introduction into the circulation of the products of putrefaction is followed by a complexus of symptoms which closely resemble what is understood clinically by the term septicæmia, and as different microbes have been cultivated from septic patients, it would seem that the disease can be produced by any of the microbes which, after their introduction into the organism, have the capacity to produce a sufficient quantity of phlogistic ptomaïnes to give rise to septic intoxication. Septic intoxication is caused by the absorption of a pre-formed ferment or toxine, which produces the maximum result as soon as it reaches the circulation, and the symptoms subside with the arrest of further supply and elimination of the septic material from the circulation.

"Septic infection, on the other hand, occurs in consequence of the introduction into the circulation of living micro-organisms which multiply with great rapidity in the blood, a circumstance which imparts to this form of septicæmia its progressive character."

"In 1857 Pasteur made the important discovery that specific micro-organisms are the cause of the various forms of putrefaction and fermentation."[6]

"Sapræmia is the typical form of septic intoxication and never occurs without putrefaction of necrosed tissue, which never occurs without infection by putrefactive bacteria. The bacilli of

[5]Senn's Principles of Surgery, p. 312.
[6]Senn's Work, p. 319.

putrefaction exercise their path[illegible]genic qualities only in dead tissue exposed to the atmospheric air."[7]

"Fetor is associated with putrefaction, and as such is suggestive of sapræmia, and not true of progressive sepsis; the most fatal form of sepsis occurs without fetor.

"Septic intoxication invariably requires three conditions, (1) dead tissue; (2) infection of this dead tissue with putrefactive bacteria; (3) a sufficient length of time must have elapsed since the injury or operation for the putrefactive bacteria to produce a toxic quantity of ptomaïnes to cause symptoms of intoxication."

"Blumberg also confirms the statement that the blood of patients dying of putrid intoxication contained no micro-organisms."[9]

"Semmer, commenting on experiments of Guttman of Dorpat, claims that a chemical poison is developed in putrefying substances, which, in sufficient quantity, produces symptoms of sepsis and death in animals. The blood of such animals possesses no infective qualities, bacteria being destroyed in the blood to appear after death. True septicæmia is always preceded by a stage of incubation, and its contagium is destroyed by boiling, *putrefaction* and germicides."[10]

Fränkel also confirms the statement that putrefaction destroys septic microbes. Brieger and Maas have rendered valuable service in the chemical isolation of ptomaïnes, or, as Brieger calls them, toxines, from putrid substances, and the results of their inoculation experiments established more firmly the fact of putrid intoxication by these alkaloid substances.

"Prof. Vaughan, Ann Arbor, divides bacteria into toxicogenic and non-toxicogenic. He further states we know of no infectious disease in which poisons are not formed, hence the toxicogenic germs only are of interest to us."[11]

---

[7] Senn's Principles of Surgery, p. 315.
[8] Senn's Principles of Surgery, p. 322.
[9] Senn's Principles of Surgery, p. 320.
[10] Senn's Principles of Surgery, p. 319.
[11] Ptomaïnes and Leucomaïnes (Vaughan), p. 13.

"Leucomaïnes are 'animal alkaloids,' those basic substances which result from tissue metabolism in the body."[12]

"Gautier includes under the name leucomaïnes all those basic substances which are formed in animal tissues during normal life, in contradistinction to the ptomaïnes or basic products of putrefaction."[13]

"Ptomaïnes are transition products in the process of putrefaction. They are temporary forms through which matter passes while it is being transformed, by the activity of bacterial life, from the organic to the inorganic state, . . . p. 16. All putrefaction is due to the action of bacteria; it follows that all ptomaïnes result from the growth of these micro-oganisms. The kind of ptomaïne found will depend on the individual bacterium engaged in its production, the nature of the material being acted upon, and the conditions under which the putrefaction goes on, such as the temperature, amount of oxygen present and the duration of the process."[14]

"Brieger points out that a certain quantity of oxygen is necessary to the formation of poisonous bases. A free supply of oxygen on the other hand, invariably yields non-toxic ptomaïnes. The poisonous bases begin to appear on about the seventh day of putrefaction, and in turn disappear, if this is allowed to go on for a considerable length of time."[15]

"The origin of leucomaïnes is indissolubly connected with the metabolism of the cell itself, and they are therefore, found in the tissues and organs proper, especially those rich in nucleated cells."[16]

From these quotations, which we have collected, representing the advance thought of the day in regard to the pathogenesis of infectious diseases, and the development of toxines, more prop-

---

[12] Ptomaïnes and Leucomaïnes (Vaughan), p. 15.
[13] Ptomaïnes and Leucomaïnes (Vaughan), p. 280.
[14] Ptomaïnes and Leucomaïnes (Vaughan), p. 17.
[15] Ptomaïnes and Leucomaïnes (Vaughan), p. 187.
[16] Ptomaïnes and Leucomaïnes (Vaughan), p. 280.

erly called ptomaïnes and leucomaïnes, we shall endeavor to present some conclusions based upon the facts quoted:

1. That puerperal infection is not due to one cause or specific micro-organism, but may be produced by the action of a variety of micro-organisms or by the effects of their alkaloidal developments.

2. That *puerperal infection* is never autogenetic but always heterogenetic in origin, either directly or indirectly.

3. That the term *puerperal septicæmia* should be applied only to puerperal morbid conditions due to entrance and multiplication of living microbes with development of leucomaïnes in the generative organs of the female and then entering the blood, producing local and systemic effects.

4. That *septic intoxication* should be used only to designate a super-saturation by septic microbes and their alkaloidal development, *i. e.*, leucomaïnes. According to the common acceptation of the meaning of *intoxication*, it should express in this connection, an overdose of sepsis.

5. That *putrid infection* should be use to designate the morbid condition resulting from the absorption of ptomaïnes developed by putrefactive bacilli.

6. *Putrid intoxication* should indicate the absorption of an excessive amount of putrefactive chemical products called ptomaïnes.

If the experiments and bacteriological researches cited in this paper are demonstrated facts, I cannot conceive how the terms *septic and putrid infection*, *septic and putrid intoxication*, can be used so indiscriminately, when we consider them in a causative relation as etiological factors in the production of puerperal complications. It is true, with our present knowledge, that it is very difficult, even impossible, in some cases to differentiate these conditions clinically, yet etiologically considered there is certainly a difference. To make this difference apparent and express it briefly, we will present in contrast some of the essential points between

| SEPTICÆMIA and | PUTRID INTOXICATION. |
|---|---|
| Due to entrance and multiplication of *microbes* and development of *leucomaïnes*. | Due to absorption of putrefactive *alkaloids* called *ptomaïnes*. |
| Due to germ life and its reproduction. | Due to absorption of chemical products. |
| Contagious. | Not contagious. |
| Inoculable from case to case. | Not inoculable. |
| Blood contains germs similar to those introduced. | Blood does not contain germs. |
| May be produced by various germs. | Produced by *ptomaïnes*, the result of putrefactive bacteria only. |
| May be produced by the fraction of a drop of the virus containing septic germs. | Requires absorption of sufficient chemical products to produce toxic effects similar to toxic doses of poisonous medicines. |
| May be produced by the virus from the exanthematous fevers. | Never. |
| Erysipelas. | Never. |
| Surgical fever. | Never. |
| A previous septic case. | Never. |
| Gonorrhœa. | Never. |

While dealing with the subject of *puerperal infection* I desire to present gonorrhœa as a causative factor in producing septicæmia, and hope to elicit discussion that will shed some light on this important question. Is it possible that gonorrhœa can produce such serious conditions of the female generative organs at other times and be devoid of influence during the puerperal state?

It cannot be successfully denied that puerperal infections are more common in the private practice of city physicians than in those practicing in the country. While a want of development of the female in the city, dress and society customs combine to produce such a result, gonorrhœa certainly plays an important part in maintaining this difference. If gonorrhœa can, under ordinary circumstances, produce endometritis, salpingitis, ovaritis, often pyosalpinx and secondarily pelvic peritonitis, then what should we expect from the presence of gonorrhœal virus and its results during abortion and labor?

If the gonococcus is not destroyed at these times by antiseptic precautions, it would certainly, at least favor the devolopment of pelvic cellulitis, pelvic peritonitis or salpingitis, with pyosalpinx, which are so often found by the gynecologist.

If gonorrhœal infection occurred previous to pregnancy from a partially cured, latent gonorrhœa in the male, which invades tissues slowly for want of activity of the virus and involves one tube, pregnancy occurring before the other tube becomes involved would favor puerperal complications.

The physiological development of the tube during pregnancy certainly furnishes a congenial soil for the development of the gonococcus and its results. Pus forms in the tube, labor occurs, and what may happen especially if the abdominal extremity of the tube be permeable? Again, suppose the gonorrhœal virus be introduced a short time previous to abortion or labor, finding lodgment in the vaginal canal and after the uterus is emptied, proceeds to invade new territory, what result should we expect?

If pus tubes occur so frequently after abortion and labor, as stated by gynecologists, there is certainly some cause for it outside of the mere process of emptying the uterus of the products of conception. I am satisfied that one of the most potent causes of this difference is gonorrhœa. If not, why are so many pus tubes found following abortion and labor occurring in the city and so few occurring in the country. No one should doubt the greater frequency of gonorrhœa in the city.

To illustrate: "Sänger, of Leipsic[17], found a purulent discharge in 100 out of 389 pregnant women, 26 per cent., and forty of the children ultimately born had ophthalmia neonatorum."

O. Oppenheimer, of Heidelberg[18], found the specific diplococcus in thirty out of 108 pregnant women, 27.7 per cent., and ophthalmia neonatorum occurred in 12 to 13 per cent.

Somer, in Schröder's clinic, of Berlin, found the specific diplococcus in nine out of thirty-two pregnant women.

Barton Cook Hirst, under "Complications of the Puerperal State," says: "The poison of gonorrhœa can, without doubt, excite inflammation of the deeper tissues in this region, and is quite certain if it spreads through the tubes to light up a sharp attack of peritonitis. The diagnosis can of course be made with approximate certainty if the disease existed during pregnancy, or if a careful examination detects an inflammation of the urethra and the vulvo-vaginal glands, or if it is possible to isolate the gono-

[17] Gonorrhœa in Women (Sinclair, W. J.), p. 283.

[18] Gonorrhœa in Women (Sinclair, W. J.), p. 283.

coccus. The consequences of gonorrhœa in the puerperal state can be of the most serious nature. There may be a rapid accumulation of pus in the tubes in the course of this disease during the puerperium."[19]

It is evident that puerperal infection is not always due to one and the same germ nor one and the same cause.

We may have puerperal infection from death, retention and decomposition of the foetus *in utero;* concealed hemorrhage with retention and decomposition; retention, decomposition and absorption from any of the products of conception after abortion or labor; *also in those cases where a cough immediately follows delivery, where genitalia are not kept properly cleansed and disinfected;* besides the ordinary channels of infection from physician, instruments or nurse, and in those cases in which the virus exists in the genital canal previous to parturition. Some of these conditions would seem to be autogenetic in origin, but not so. The dead foetus, concealed hemorrhage, retained maternal or foetal structures cannot of themselves infect the patient until changes take place in them not wrought by powers inherent to themselves nor generated by the mother, but originally received from other sources. This is also true of gonorrhœa and pyosalpinx as etiological factors, for the infectious virus was originally generated elsewhere and transmitted to the mother.

Puerperal infection may be divided into two general classes as to contagiousness or transmissibility from case to case. It is certainly an axiom that those cases due to the entrance of septic germs and their development in the blood and various organs of the body are contagious, transmissible and may be easily conveyed or inoculated from case to case.

Not so with those cases due to a chemical product, the result of putrid decomposition. Such cases are not inoculable or transmissible from case to case, only when a sufficient amount of the chemical product of decomposition—ptomaïne—is transmitted to produce its effects the same as a toxic dose of poisonous medicine. It may be indirectly transmissible by conveying the vagi-

[19] Am. Sys. of Obstetrics, p. 522.

nal secretions from a case of putrid intoxication to the vagina or uterus of a recently delivered woman where the uterus or vagina contains dead foreign material, thus instituting putrid decomposition of such retained material, developing ptomaïnes, which are absorbed, affecting the patient.

Hence the necessity for strict cleanliness under all circumstances, and under some conditions the almost imperative demand for *antiseptic precautions.* The physician is not responsible for all cases of puerperal infection, but for a majority at least, and should use all reasonable, rational means at his command to prevent infection.

If our basis and conclusions be correct as to the etiology and transmissibility of puerperal infection, then such expressions from a physician in active obstetric practice for ten or fifteen years, "I never have any cases of puerperal fever," *is but the confession of ignorance or a misstatement of the facts.* It is but another example of the obstetrician who, in fifteen or twenty years' practice, never encountered a lacerated perineum, *while the gynecologist was continually following his work repairing the perineum.* So to-day the gynecologist is often called upon to treat the results of puerperal infection. Some physicians never have a case of puerperal infection until their patient dies, and then the death certificate records a death from "inflammation of the bowels," "malarial fever," "typhoid fever," etc., etc.

The mortuary statistics on this subject are very unreliable as a rule, and worth but very little from a scientific standpoint. The idea becoming prevalent among the profession and the laity to a great extent that the physician was responsible for all cases of puerperal infection occurring in his practice, the physician, as a natural result, would use his diagnostic skill to class all such cases under some name for which he would not be held responsible. Then again the death rate thus reported has been taken as a guide to the number of cases of puerperal infection occurring, and as an *indication* or *contraindication* for the use of *antiseptics.*

In an article read before the Indiana State Medical Society in 1888 by Dr. L. N. Davis, on the "Obstetric Record of the

Country Doctor," we find a record of 3,752 cases of obstetrics, with only seven deaths from "puerperal fever."

Dr. G. W. H. Kemper reported next year 900 cases, with no deaths from "puerperal fever," thus making 4,652 cases, with seven deaths, or one in every 665 cases. "No antiseptic precautions taken in the practice of these physicians," so reported. It is not my province to call in question these statistics, as they are vouched for by *competent*, *honorable* men, especially Dr. G. W. H. Kemper, an ex-president of the Indiana State Medical Society. They undoubtedly show an excellent record, and lead us to inquire why this difference between city and country obstetric practice? The record of these six physicians, whose ability would render them competent to conduct labor aseptically, if not antiseptically, should not be taken as a correct guide as to the death rate in country practice, where all classes of physicians attend cases of labor.

I wish to offer a protest against making the death rate a basis on which to calculate the number of cases of *puerperal infection, or as an indication or contraindication for the use of antiseptic precautions.* When we recognize that puerperal infection may produce any of the following named conditions, vulvo-vaginitis, endometritis, salpingitis, uterine lymphangitis (most frequent condition resulting from infection), peritonitis (pelvic and diffused), adeno-phlegmon (inflammation of lymphatic glands and surrounding connective tissue, called pelvic cellulitis and parametritis), and phlebitis (adhesive and infectious); that any of these conditions may be produced in a light or severe form according to amount, quality and activity of virus introduced, as well as the condition of the patient and organs infected; that the infecting virus may be developed from such a variety of sources, and conveyed in so many different ways, as previously alluded to in this paper; that the effects of infection are not always wrought immediately, but often result in subinvolution, inflammatory deposits, fixation of uterus and appendages in abnormal positions and pyosalpinx. Then we are in a condition to fully realize the import of the term *puerperal infection*, and see the necessity of adopting means to prevent these various conditions, originating primarily from so many various sources.

